# The acceptability, usability, engagement and optimisation of a mHealth service promoting healthy lifestyle behaviours: A mixed method feasibility study

**DOI:** 10.1177/20552076241247935

**Published:** 2024-04-17

**Authors:** Callum Regan, Phillip Von Rosen, Susanne Andermo, Maria Hagströmer, Unn-Britt Johansson, Jenny Rossen

**Affiliations:** 1 Division of Physiotherapy, Department of Neurobiology, Care Sciences and Society, 27106 Karolinska Institutet, Stockholm, Sweden; 2 Division of Nursing, Department of Neurobiology, Care Sciences and Society, 27106 Karolinska Institutet, Stockholm, Sweden; 3 Department of Sport Science, The 42750 Swedish School of Sport and Health Sciences, Stockholm, Sweden; 4 Academic Primary Health Care Centre, 7674 Region Stockholm, Stockholm, Sweden; 5 Department of Health Promoting Science, 156271 Sophiahemmet University, Stockholm, Sweden; 6 Department of Clinical Science and Education, Södersjukhuset, 27106 Karolinska Institutet, Stockholm, Sweden

**Keywords:** Acceptability, behaviour change, engagement, feasibility studies, lifestyle, mHealth, mixed methods, mobile applications, public health and usability testing

## Abstract

**Objective:**

Mobile health (mHealth) services suffer from high attrition rates yet represent a viable strategy for adults to improve their health. There is a need to develop evidence-based mHealth services and to constantly evaluate their feasibility. This study explored the acceptability, usability, engagement and optimisation of a co-developed mHealth service, aiming to promote healthy lifestyle behaviours.

**Methods:**

The service LongLife Active® (LLA) is a mobile app with coaching. Adults were recruited from the general population. Quantitative results and qualitative findings guided the reasoning for the acceptability, usability, engagement and optimisation of LLA. Data from: questionnaires, log data, eight semi-structured interviews with users, feedback comments from users and two focus groups with product developers and coaches were collected. Inductive content analysis was used to analyse the qualitative data. A mixed method approach was used to interpret the findings.

**Results:**

The final sample was 55 users (82% female), who signed up to use the service for 12 weeks. Engagement data was available for 43 (78%). The action plan was the most popular function engaged with by users. The mean scores for acceptability and usability were 3.3/5.0 and 50/100, respectively, rated by 15 users. Users expressed that the service’s health focus was unique, and the service gave them a ‘kickstart’ in their behaviour change. Many ways to optimise the service were identified, including to increase personalisation, promote motivation and improve usability.

**Conclusion:**

By incorporating suggestions for optimisation, this service has the potential to support peoples’ healthy lifestyle behaviours.

## Introduction

Mobile health (mHealth) is the use of mobile technologies to achieve health outcomes.^1^ It is estimated that there are 4.6 billion smartphone users, globally.^2^ Sweden is one of the most digitalised countries in the world,^3^ with 78% of the population using a smartphone and having access to the Internet.^4^ In this technological day and age, given how resource-intensive in-person healthcare is,^5^ there is a need for inexpensive services which promote healthy lifestyle behaviours that are suitable for modern-day lifestyles.^6^ mHealth services can provide information at any time, reach large populations and be more cost-effective than in-person healthcare.^7^ When developed appropriately, mHealth services can assist behaviour change by raising health awareness, self-monitoring, providing feedback and increasing motivation.^8^ Notably, mHealth services can provide personalised support for managing healthy lifestyle behaviours.^9^ Therefore, mHealth services represent a significant strategy to promote and support healthy lifestyle behaviours.

mHealth interventions which incorporate frameworks of behaviour theory, such as the Transtheoretical Model of Change and the Social Cognitive Theory, have been shown to facilitate behaviour change.^10,11^ Interventions that incorporate behaviour theory are more effective for eliciting behaviour change than those that do not.^11,12^ Moreover, mHealth interventions that incorporate behaviour change techniques (BCTs) have a higher efficacy in achieving health behaviour change in multiple lifestyle behaviours, than those that do not.^13^ Self-monitoring and goal setting have been shown to play a fundamental role in achieving health behaviour change,^14^ as well as receiving both feedback^13,15^ and human coaching.^16^ However, the use of behaviour theory and BCTs in mHealth services is not always reported,^10^ nor evaluated,^12^ thus it can be unclear what in a mHealth service facilitates behaviour change. This ambiguity can be minimised by linking BCTs to the functions in mHealth services and measuring user engagement levels of each function.

Incorporating the perspectives of researchers, healthcare professionals and product developers, in a co-development manner, has been suggested to be important for the implementation of mHealth services.^8^ This collaboration can allow mHealth services to become developed on evidence-based research. Moreover, perspectives from user experiences can provide valuable information for the development and optimisation of mHealth services.^17^ Users have highlighted the importance of mHealth services to be personalised,^18,19^ varied,^15,18^ provided by a credible source,^16^ reliable and easy to use.^18^ Users also have valued the use of push notifications, especially if personalised.^18^ The incorporation of evidence-based research, through co-development, can increase the credibility of information provided by mHealth services. This and the incorporation of users’ perceptions, can, therefore, allow mHealth services to be more suitable and effective for their respective target population, simultaneously reducing research waste.

A major challenge of mHealth services is overcoming high attrition rates,^20^ partly due to low intrinsic motivation^21^ and concerns over information quality and data security.^22^ Most mHealth services lead to short-term health behaviour changes, rather than long-term behaviour maintenance.^23^ It has also been found that mHealth interventions in general adult populations have produced mere modest improvements in health behaviour outcomes.^10,13,24^ It is, thus, paramount to identify users’ reasons for engagement and barriers to use,^21^ yet limited evidence exists regarding the engagement of mHealth services.^13,25^ Moreover, there is limited evidence regarding the acceptability and usability of mHealth services,^25^ which can provide reasons for engagement and barriers to use. The lack of feasibility testing could be an explanation for the high attrition and low efficacy rates observed. Therefore, there is a need to incorporate evidence-based research in mHealth services, whilst evaluating their feasibility and exploring users’ views on how to optimise such mHealth services. Behaviour change interventions can be optimised based on user feedback; ensuring that the intervention can be as meaningful, feasible and engaging as possible.^26^

LongLife Active (LLA) is a mHealth service that aims to promote and support healthy lifestyle behaviours in adults, specifically those who want to change their behaviour. LLA is a mobile app that also provides a digital coaching service. LLA was developed through a co-development process and grounded in evidence and theory-based research. This feasibility study aimed to explore the acceptability, usability, engagement and optimisation of LLA, in users from a general adult population, as well as in product developers and coaches of LLA.

## Methods

### Study design and setting

This study represents the feasibility stage of the development of a complex intervention, as laid out in the Medical Research Councils’ frameworks for developing and evaluating complex intervention design.^17,27^ This feasibility study aimed to inform on the development of LLA. The study was approved by the Swedish Ethical Review Authority (Dnr: 2021-04287).

The LLA mHealth service is a mobile app and coaching service that was delivered digitally, free of charge, to users. Users were recruited during February–March 2022. All users then had access to LLA, at the same time, from April to June 2022; encompassing the 12-week study period. It is intended that LLA can be used flexibly by users and the extent of engagement needed to elicit benefits for behaviour change is different for different users, as suggested in Donkin et al. (2011).^28^ As goals are likely to vary among users,^29^ users are not required to engage in all of the available functions in LLA. Therefore, during the study period, users could use the app as much or as little as they wanted and users were not asked to try out any specific function of the app.

The study design is based on a framework for developing mHealth interventions^30^ and used both quantitative and qualitative data to investigate the feasibility of LLA. A convergent parallel mixed method design was used, where quantitative data and qualitative findings were performed and analysed separately,^31^ and integrated when interpreting the results. The qualitative component complemented and expanded on quantitative results.^32^ Both quantitative results and qualitative findings guided the reasoning for the overall acceptability, usability, engagement and optimisation of LLA. The Consolidated Criteria for Reporting Qualitative Research (COREQ) checklist was followed^33^ (Supplementary file 1).

### Participants and recruitment

**Figure 1.**
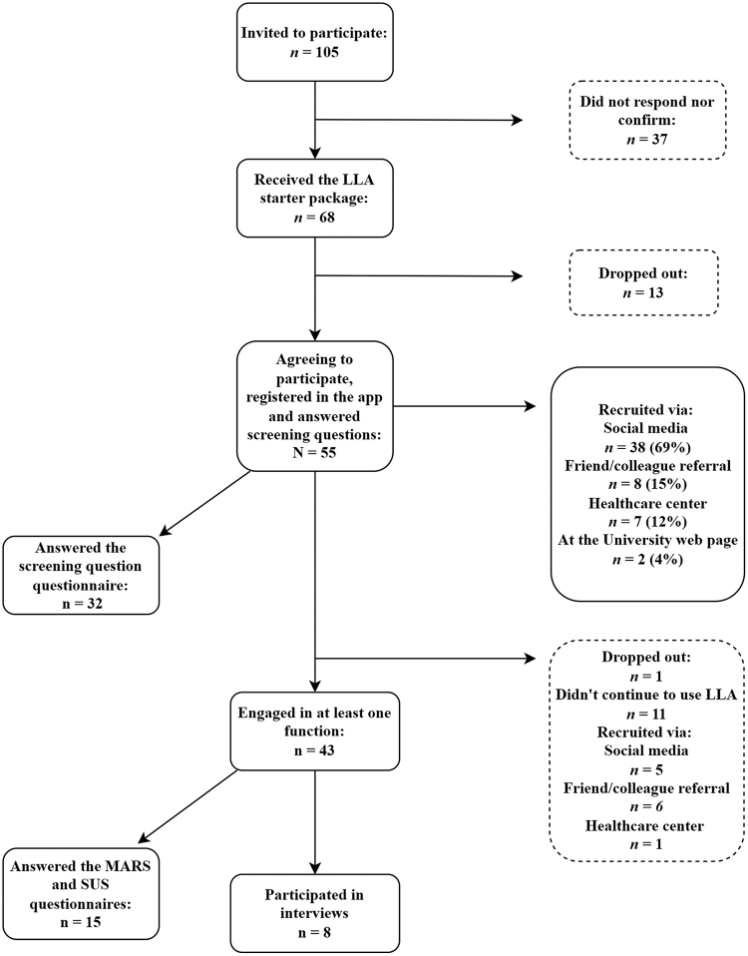
Flowchart showing recruitment of users.

The inclusion criteria for users were: at least 18 years of age, having access to a mobile smartphone and Internet and being able to understand Swedish. Users were recruited from the general population, through a non-randomised convenience sampling method. Users were recruited through: advertisements on social media (Facebook), referrals by a colleague or friend, flyers at healthcare centres and advertisements on Sophiahemmet University's web page. A flow chart for user recruitment and drop-outs is shown in Figure 1.

Information about the study was sent via email to all users before participation. A starter package about LLA was also sent via post to all users before participation. The starter package included information on study dates, how to download the app on Android and iOS mobiles, the content of LLA and how to contact the researchers. Exercise bands of varying resistance and a paper calendar for action planning were also provided in the starter package.

All users provided informed consent to be part of the study in the app when they signed up to use the service. All users were invited to be part of the interviews, through a message invitation in the app. Several users responded to the message invitation, the remaining users were conveniently sampled to participate in the interviews via an invitation through email or a telephone call, from one of the product developers of LLA. Potential interviewees were provided information on data anonymity and gave verbal consent to be part of the interviews before the interviews started.

Product developers (the designers and creators) and coaches (who completed a 3-year bachelor's degree in health education) were employed by and recruited through the company LongLife Active^®^. Product developers and coaches provided consent to be part of the focus groups and for the focus groups to be recorded. Participation was voluntary and any user, product developer or coach could drop out at any time. Data storage of quantitative and qualitative data ensured the users’ anonymity.

### Development of the LLA mHealth service

LLA is a commercially available mobile app that provides digital coaching, intending to promote and support physical activity (PA), healthy eating and mental balance lifestyle behaviours. The target population of the app version tested in this study was any adult in the general population, as it was thought that the service could be suitable for any adult who wants to improve or needs support with their lifestyle behaviours. LLA has been designed for those with low digital literacy or low levels of the Swedish language with easy-to-use functions and illustrations. LLA uses artificial intelligence to personalise preferences to the individual user. LLA was designed and developed by the company LongLife Active AB^®^ through a co-development approach, involving consultation and collaboration with researchers and stakeholders, as suggested in Yardley et al. (2015).^30^ The collaboration started in 2021 and included stakeholders of multiple research and healthcare institutions, including Sophiahemmet University, Karolinska Institutet, Linnaeus University, The Swedish School of Health and Sport Sciences, Research Institutes of Sweden, Södertörn University, Stockholm University and Academic Primary Care Centre Region Stockholm. Researchers, product developers and coaches employed by LLA, and healthcare providers had a total of nine design workshops before the pilot version was tested. Motivation and engagement were chosen as the core guiding principles for the development of the service. The content and structure of the app and which BCTs could be integrated were discussed and brainstormed during these workshops.

The development of LLA was based on evidence from several studies, carried out by members of the co-development team, including a systematic review^18^ and two qualitative studies.^34,35^ The purpose of these studies was to identify what users desire in mHealth services and what users desire for behaviour change support. LLA was also developed with behavioural theory and functions in the app were linked to BCTs (Supplementary file 2) using The Behaviour Change Technique Taxonomy (v1).^36^ Adaptations of functions were guided by the Transtheoretical Model of Change.^37^

### Functions in LLA

LLA has three core areas: (1) PA, (2) healthy eating and (3) mental balance. Functions included: action plan (goal and activity setting), knowledge library, community, coaching and feedback (Supplementary file 3). Users could also answer screening questions when they signed up for the service. Screening questions asked users about their lifestyle habits and aimed to assist users by providing suggestions for goals and activities. This assistance was done with the aid of artificial intelligence.

The action plan is used to set goals and activities within the three core areas. The difference between goals and activities is that activities acted as a means to achieve goals and were set daily to achieve weekly goals. Thus, after setting a goal, activities were then suggested to help achieve that particular goal. Examples of goals included: eating two vegetarian meals in a week or completing three PA sessions in a week. Examples of activities included: going for a brisk walk every Monday and Thursday or doing yoga on a Friday. Goals and activities could be edited and changed at any time and were visualised as a weekly programme. After a goal or an activity was set, users could register to complete that particular goal or activity. Users did not receive reminders to complete goals or activities. However, when either goals or activities were completed an encouraging pop-up message appeared on the user's screen.

The knowledge library function provided information based on research regarding PA and health (e.g., about the global recommendations to aim to achieve at least 150 minutes of moderate to vigorous exercise per week^38^), healthy eating advice (inspired by the Swedish Food-based dietary guidelines, for instance, to eat 500 g of fruit and vegetables every day^39^), sleep and overall well-being. Video-recorded PA exercises, food recipes and tips on overall well-being were also included in the knowledge library.

The community function included weekly inspirational and educational content for the users to engage with. Weekly content was uploaded by product developers. Users could answer quizzes and make comments on the uploaded content. Users could also engage with each other, by responding to each other's comments.

The coaching function allowed participants to book individual or group digital coaching sessions with trained coaches. Users could receive support regarding their action plans. The main focus of these coaching sessions was to address the behaviour change required to carry out desired healthy lifestyle behaviours. Each user could use the individual coaching service up to three times and take part in all four of the available group coaching sessions.

Functions in LLA were updated five times during the study period, by product developers. The updates occurred in weeks: 1, 4, 7, 9 and 11. Updates included: improving accessibility to one's profile page and to leave feedback, daily inspirational quotes in the action plan, automatic email reminders for booked coaching sessions, allowing re-scheduling of coaching sessions if they were missed, creating a lectures section with a “learn to cook” series in the knowledge library, and allowing for push-notifications for daily reminders to use LLA when users had set goals. The updates did not drastically change the version of the app, were based on feedback from users and were completed during the study period to mimic real-world settings.

### Data collection

#### Demographic characteristics

Demographic information (age, gender, country of birth and education status) of the users was obtained via an online questionnaire. Screening questions were answered by users when they started using the service. The purpose of the screening questions was to gather information on users’ healthy lifestyle behaviours (from the three core areas: PA, healthy eating and mental balance). Screening questions included –How many minutes of PA do you do weekly? How many minutes of PA that gets you out of breath do you do weekly? How often do you eat fruit and vegetables? Do you feel that you can relax from thoughts of worry and stress? Answers relating to PA were used to assess the proportion meeting the PA guidelines^40^ (at least 150 minutes of PA or at least 75 minutes of PA that gets you out of breath, per week) and answers from the fruit and vegetable question were used to categorise users into daily consumption or not. Demographic information for product developers and coaches was not collected.

#### Acceptability and usability

Acceptability, encompassing satisfaction, suitability and perceived appropriateness,^41^ was measured via the Mobile Application Rating Scale (MARS)^42^ (Supplementary file 4) and qualitative findings. MARS was translated into Swedish and adapted to tailor questions to the LLA service (the word ‘app’ was replaced with ‘service’). MARS measures overall app quality and consists of 19 items, encompassing four main themes: engagement (A), functionality (B), aesthetics (C) and information quality (D).^43^ There are also two additional themes: overall app subjective quality (E) and perceived impact on users’ knowledge, attitudes and intentions for behaviour change (F). Items are answered on a 5-point scale and answers range from ‘inadequate’ to ‘excellent’.^43^ A mean score is taken from sections A–D, ranging from 0 to 5, to measure the overall quality of the app^42^; higher scores indicate higher ratings of quality. A mean score of ≥ 3 was deemed to be acceptable.^43^

Usability, encompassing efficiency and satisfaction of using the mHealth service,^44^ was measured via the System Usability Scale (SUS)^44^ (Supplementary file 5) and qualitative findings. SUS was translated into Swedish and adapted to tailor questions to the LLA service (the word ‘system’ was replaced with ‘service’). SUS contains ten questions, which are answered on a 5-point Likert scale with answers ranging from “strongly disagree” to “strongly agree”.^44^ Scores are converted from scale positions; for odd items, the score equals the scale position minus 1 and for even items the score equals 5 minus the scale position. Mean scores range from 0 to 4 and are added from each question and multiplied by 2.5. Scores can range from 0 to 100^44^; higher scores indicate higher ratings of usability. A mean score of ≥ 70 was deemed to be acceptable and would indicate only minor changes needed for usability.^45^

Users had the opportunity to answer sections A–D of MARS and SUS from the third week. Sections E and F of MARS were available to answer from the seventh week. One response per user was used.

#### Engagement

Engagement levels were measured through app log data and qualitative findings and sought to investigate the engagement in the different functions and how engagement changed with time. Log data was measured weekly for each function, over the 12 weeks. For the action plan function, how many, which, and for how long goals and activities were set up as well as how often users registered goal and activity completion were measured. For the community function, the number of comments and when comments were written were measured. For the coaching function, how many and when users undertook sessions were measured. Log engagement data for the knowledge library were unfortunately lost; therefore, no data exists for the knowledge library function. As the extent of engagement to acquire behaviour change benefits from LLA is intended to be different for each user, there was no quantitative acceptable threshold for sufficient engagement. Moreover, as goals amongst users were likely to differ, users were not required to use all the functions in LLA to benefit from using the service. Both quantitative, including acceptability and usability scores and qualitative findings aimed to explain engagement patterns.

#### Screening question questionnaire

The screening question questionnaire was available in the first week of the study and aimed to understand how well the screening questions (asking about users’ lifestyle habits) helped users set up goals. Questions included: Was it simple to answer the screening questions? Were the questions relevant to your lifestyle? How well did you feel the service helped you concrete your goals? How does it feel to leave personal and sensitive data in the service? These were rated on a 5-point scale, with answers ranging from ‘not at all simple/relevant/well’ to ‘very simple/relevant/well’. Mean scores were used to represent the results. Scores ≥ 4 were considered acceptable.

#### Interviews, focus groups and feedback

Eight semi-structured interviews were conducted among users, which were held in Swedish. The purpose of the interviews was to explore users’ experiences of using LLA, perspectives on acceptability, usability and engagement and suggestions for how LLA could be optimised, as suggested in Morrison et al. (2018).^26^ The potential influence of the service in helping users with their behaviour change was also explored during the interviews. Interviews were conducted by one of the two researchers – SA and JR. SA is an assistant professor with a master's degree in public health (PhD). JR is a senior lecturer with a master's degree in nutrition (PhD). SA and JR have extensive experience in conducting interviews and focus groups and had no prior relationship with the users before the study. One product developer was also present for the interviews to clarify any technical aspects of the app and to probe questions regarding app optimisation. The product developer took part in interviews to hear first-hand feedback on LLA, which would facilitate the development of LLA, as part of the co-development process. Users were informed that the researchers wanted to explore their experiences of using LLA and their suggestions for optimisation. Interviews were performed after the 12 weeks (September 2022), digitally on Zoom. An interview guide, developed by the product developers and researchers, was used to steer questions during the interviews. The interview guide was not pilot tested, however, after the first interview no major concerns over the interview guide arose. Questions included: How did you experience the service overall? Did the service inspire and motivate you to create healthy lifestyle habits? Explain how the service inspired and motivated you to create healthy lifestyle habits or how it did not. How did you experience LongLife Active's presentation and approach to well-being and health? How did you experience setting and following up on goals in the area of ​​ PA? Interviews were audio and video recorded. The recorded interviews lasted between 30 to 75 minutes. Transcripts were not returned to users.

Two focus groups were conducted, led by SA and JR. The purpose of the focus groups was to investigate product developers’ and coaches’ experiences of operating LLA, perspectives on acceptability, usability and engagement, as well as their suggestions on how LLA could be optimised. The first focus group was conducted with two coaches and two product developers and was held in Swedish. Product developers were included in the focus group with the coaches to explore if they both shared similar experiences regarding working with LLA and to discuss the benefits and challenges of being part of the co-development process. The second was conducted among four product developers of LLA and was held in English. Focus group discussions were performed after 12 weeks (August and September 2022), digitally on Zoom. The interview guide was adapted for the focus groups. Questions included: How has your experience been working on the overall development of the service? How did you feel that the service as a whole worked over the study period? What challenges do you see in developing a digital health service that aims to motivate long-term behaviour change? What do you think should be prioritised in the future development after the study? Focus groups were audio and video recorded. The recorded focus groups lasted between 105 to 120 minutes. Transcripts were not returned to product developers or coaches.

Through the app, ten users’ feedback also contributed to qualitative findings. Users could write or send voice messages of feedback regarding their experiences of using LLA and suggestions for optimisation via the feedback function in the app. Users could also send feedback to the LLA support email and vocally express feedback during the coaching sessions. All users could leave feedback continuously during the 12 weeks.

### Data analysis

Descriptive statistics were used to present users’ demographic information, acceptability, usability, engagement levels and screening question questionnaire responses. Mann–Whitney *U*-tests were used to compare engagement data between those with at least one coaching session and those without. SPSS version 28.0.1.1, IBM, New York, USA and Microsoft Excel, Microsoft Corporation, Washington, USA, were used to present descriptive data. SPSS version 28.0.1.1 was used to carry out Mann–Whitney *U*-tests.

Qualitative data from interviews and the first focus group (230 pages in total) were transcribed by a professional. CR transcribed the second focus group (25 pages). Qualitative data from interviews, focus groups and feedback were analysed using manifest content analysis^46,47^ with an inductive approach.^48^ All materials were used as the unit of analysis. Meaning units, relevant parts of the transcript to be analysed, were produced and condensed. Appropriate codes, from meaning units, were created from each interview/focus group/feedback by CR and validated by JR. All codes were brought together before themes and sub-themes were derived from the data. All authors discussed and agreed on the themes, sub-themes and quotations representative of the results, following procedures in Graneheim and Lundman, 2004.^47^ A coding tree illustrates the use of this approach (Supplementary file 6). SA has considerable experience in conducting content analysis, whilst JR and PVR have some experience and CR limited experience. Users, product developers and coaches were not asked to provide feedback on the findings. Microsoft Excel was used to manage the data.

## Results

### Demographic characteristics

A final sample of *N* = 55 users agreed to be part of the study and use the LLA service for 12 weeks (Figure 1). Forty-five (82%) of the users were female, 39 (71%) were between the ages of 50 to 64, 52 (96%) were born in Sweden and 25 (45%) had an education beyond high school (Table 1). Demographic characteristics of the interview sub-sample (*n* = 8, 15%) are also shown in Table 1. The interview sub-sample had a higher proportion educated at University/College level than the main sample (Table 1).

**Table 1.** Users’ characteristics who signed up to use the service.

Characteristics	Users (*n* = 55)	Interview sub-sample (*n* = 8)
	*n* (%)	*n* (%)
**Gender**		
Female	45 (82)	6 (75)
Male	9 (16)	1 (12)
Other	1 (2)	1 (12)
**Age (years)**		
18–29	1 (2)	0 (0)
30–49	7 (13)	2 (25)
50–64	39 (71)	6 (75)
≥65	8 (14)	0 (0)
**Place of birth**		
Sweden	52 (95)	8 (100)
EU	2 (4)	0 (0)
Outside EU	1 (1)	0 (0)
**Education level**		
High school	30 (55)	2 (25)
University/ College	25 (45)	6 (75)

The final sample (*N* = 55) answered screening questions in at least one core area; *n* = 40 answered questions relating to PA, *n* = 26 answered questions relating to healthy eating and *n* = 21 answered questions relating to mental balance. Answers from the screening questions included: 16 users (40%) met the PA guidelines, 23 users (89%) reported daily fruit and vegetable consumption and 16 users (76%) reported dedicating time to recover from everyday life. Answers relating to lifestyle habits, from screening questions, were proportionally similar between the total sample (*n* = 55) and the interview sample (*n* = 8) and between the total sample and the sample that engaged with at least one function in the service (*n* = 43) (results not shown).

### Acceptability and usability

The overall mean MARS score, measuring acceptability, was 3.3 out of 5.0 (rated by *n* = 15). The mean score for each section of MARS can be seen in Table 2. The overall mean SUS score, measuring usability, was 50 out of 100 (rated by *n* = 15).

**Table 2.** Mean, min and max scores for the MARS sections (out of 5), for *n* = 15, unless specified.

MARS Sections	Mean	Min.	Max.
Engagement (A)	3.0	1.0	4.6
Functionality (B)	2.8	1.3	4.0
Aesthetics (C)	3.3	1.7	4.7
Information quality (D)	4.0	1.8	5.0
App subjective quality^a^ (E)	2.7	2.5	3.0
Perceived impact on user's knowledge, attitudes and intentions for behaviour change^a^ (F)	2.5	1.2	3.7

^a^ For *n* = 4.

### Engagement data

**Figure 2.**
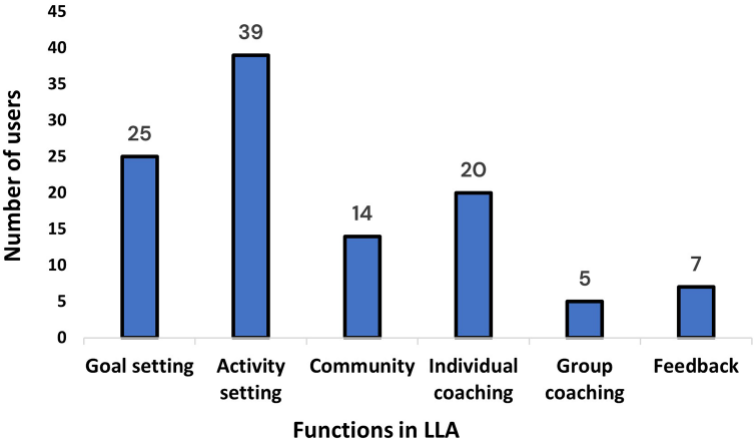
Number of users that engaged with the app functions, at least once, out of those who engaged in at least one function, *n* = 43.

Out of the total sample, *n* = 43 (71%) engaged with at least one function in the app at least once during the 12 weeks (Figure 2). Therefore, *n* = 12 (19%) users either answered the screening questions and then did not continue to use the app or answered the screening questions and only used the knowledge library function (data were lost for the library function).

**Figure 3.**
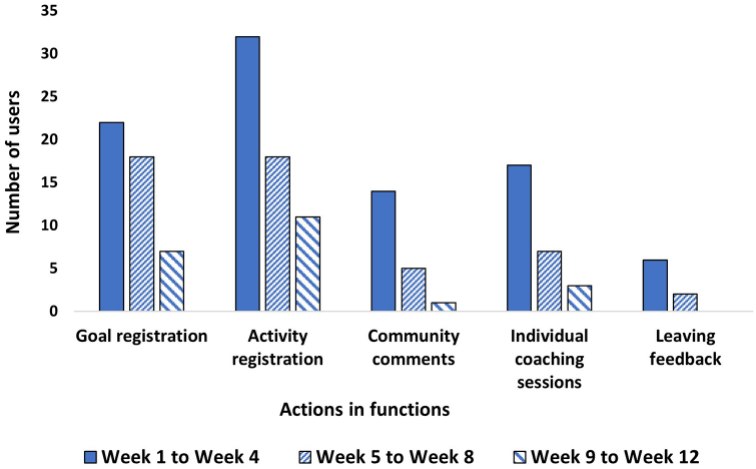
User engagement of performing actions in functions, *n* = 43.

Regarding goal setting, *n* = 25 (45%) set goals during the 12 weeks (Figure 2). Of these *n* = 25, *n* = 13 (52%) set one goal whilst *n* = 12 (48%) set two or three goals. PA was the most popular area where users set goals, followed by mental balance and healthy eating. Most users set goals in just one of these areas, with only three users setting goals in all three areas. More goals were set earlier in the study period compared to the latter weeks – the same trend held for goal registration, as shown in Figure 3. Concerning activities, *n* = 39 (71%) set activities during the 12 weeks (Figure 2). PA was the most popular area where users set up activities, followed by mental balance and healthy eating. More activities were set earlier in the study period compared to the latter weeks – the same trend held for activity registration (Figure 3).

For the community function, *n* = 14 (25%) used the community function during the 12 weeks (Figure 2). A total of 38 comments were written in the community function with 33 having been written between weeks 1 and 4. The number of users engaging in the community function across the 12 weeks is shown in Figure 3. The median number of comments per user was two, with a minimum of one and a maximum of seven.

Regarding the individual coaching sessions, *n* = 20 (36%) partook in digital coaching during the 12 weeks (Figure 2). Out of those who partook in the digital coaching, *n* = 15 (75%) users had just one individual coaching session. There was a total of 27 coaching sessions, 17 (63%) of which took place between weeks 1 and 4 (Figure 3). It was shown that those who had at least one individual coaching session and engaged with the action plan, *n* = 16, set significantly more goals (p < 0.01) and set activities for a significantly longer time (p < 0.01) than those who engaged in the action plan but did not have any individual coaching, *n* = 23. Moreover, although not significant, those who had at least one individual coaching session and engaged in the action plan tended to set goals for a longer time and engaged more in goal registration compared to those who engaged in the action plan but did not have any individual coaching.

There were two group coaching sessions, with *n* = 5 (9%) users. Three users took part in the session on PA and two users took part in the session on mental balance. There were two other group sessions available on healthy eating and behaviour change, but no users participated. Furthermore, concerning the feedback function, *n* = 7 (13%) wrote comments during the 12 weeks (Figure 2), whilst *n* = 3 (5%) provided feedback through the support email. In the feedback function, nine comments in total were written, most of which were written between weeks 1 and 4 (Figure 3).

### Screening question questionnaire

The screening question questionnaire was answered by *n* = 32 (58%) users. Mean values out of 5 were used for ratings. Users thought the questions were simple to answer (rated 4), thought the questions were relevant to their lifestyle (rated 4), thought that the service was ok at helping them concrete their goals (rated 3) and felt good to leave personal information (rated 4).

### Interviews, focus groups and feedback

#### Themes

Qualitative findings, from interviews, focus groups and feedback elucidated three major themes and two sub-themes. The three major themes were as follows: ‘One size does not fit all – views on appreciation, suitability and personalisation’, ‘Influencers of engagement’ and ‘The service promoted health awareness and assisted behaviour change’. Two sub-themes emerged under the theme ‘Influencers of engagement’ and were as follows: ‘Facilitators of engagement’ and ‘Barriers of engagement’.

### One size does not fit all – views on appreciation, suitability and personalisation

Most users enjoyed using the different functions, found them to be suitable for their health and had a positive experience of using the service. Some found the screening questions to be easy to answer, important and suitable for their health. The action plan and coaching in particular were appreciated.

I like the checklist system [in reference to the activities in the action plan]; I like this here when you can click when it is done. – Study ID 124

Users found the content to be inspirational in the library function and some became inspired to learn new information.

Yes, but I liked them [in reference to the articles in the library function], they really follow me, so, so it was a great arrangement to read them so, mm. – Study ID 154

The language delivery of the content was also deemed to be appropriate. Some users expressed that the action plan and coaching were tailored to suit their needs. Moreover, several users thought that the service filled a function and the app itself could be used as a health coach.

Coaches voiced that the coaching sessions went unexpectedly well and surpassed their expectations. They too found that users appreciated the sessions. Coaches felt that it was rewarding and comfortable to give support. One coach voiced that the digital format of the coaching session allowed the coach to come close to the user, by feeling that they played a part in the user's everyday life. However, coaches found that users could become distracted by other household duties, during the digital coaching sessions and thought that this reduced the value of the sessions.

Product developers and coaches believed that the service provided a lot of value.

It feels like we are providing so much value within the app. – Product developer ID 3

Moreover, product developers thought that using research evidence could help create trustworthiness with the users. Product developers and coaches also thought that: support from coaching, the community function and personalisation were crucial components in helping users with their lifestyle habits.

However, several users expressed feeling disappointed and that the service did not meet their expectations. Some felt that the screening questions and goal recommendations did not suit their needs whilst others found that the PA exercises, recipes and the content of mental balance were not suitable for them. Several users too expressed that the service was not particularly special and did not give much inspiration.

I didn’t find the inspiration, so what I hoped for was that I would get inspiration from this, your app. – Study ID 156

Additionally, one user found that the use of the mobile phone was stressful, however, this person stated that using the starter package and the app was a good variation.

Additionally, users suggested optimisations for content that would be appreciated. Suggestions included: new recipes and PA exercises, more variation in recipes, more convenient ingredients for recipes, a larger section on sleep and a section providing advice on acquiring and maintaining a healthy work-life balance.

Some users expressed that more personalisation was needed. Optimisations for personalisation included: personalised notifications and tips, being able to set how many notifications one receives and more flexible goal setting and registration – including being able to write your own specific goals and register specific trainings and training intensities.

Be able to register activities afterwards, as well as be able to enter, for example, vegetables twice a week without specifying which days. – Study ID 139

Furthermore, coaches expressed a need to improve the usability of the coaching function, from their booking view, when organising and performing the coaching sessions. These included: a view to see bookings, the ability to set availability for coaching sessions, a note-taking function and functions integrated into the app — instead of having to leave the app to carry out particular tasks.

### Influencers of engagement

#### Facilitators of engagement

Several users expressed that usability facilitated engagement. These users thought that the service was technically simple, practical to use, easy to navigate and visually appealing. In particular, booking the coaching sessions was found to be simple and functional.

It was easy to book these times with the coach, eh and the connection…ehh that I thought was good, it worked well. – Study ID 156

It was also voiced, by a few users, that the whole layout of the service was clear to understand and a solid foundation had been built.

Users expressed other factors that facilitated engagement, including inner motivation, timing and the convenience of having the service on a mobile phone. A few users also found it exciting to be part of a research study and engaged because they wanted to advise the service.

Nevertheless, users expressed several optimisations that could facilitate engagement. A need for more push and motivation was voiced. Suggestions for motivation included more: notifications, reminders, rewards, follow-ups and feedback summaries on goal progress (with the use of statistics and charts).

Think there should be a function in the app that reminds you that “now is the time” to register or accomplish today's goals. – Study ID 135

Additionally, more social interaction was desired to encourage each other more in the app and having a group chat function was suggested. Several users voiced that having people you know on the service would help to facilitate engagement. It was also suggested that creating a common platform between users and coaches would allow more communication and encourage engagement in the coaching function.

Product developers found the service to be functional and technically stable. Coaches thought that the coach booking system worked smoothly and experienced only a few minor technical issues. Coaches also voiced that preparing for coaching sessions allowed them to be more engaged, which could facilitate a relationship and trustworthiness with the users, in turn encouraging user engagement.

#### Barriers of engagement

Users voiced usability difficulties as focal barriers of engagement. Navigation difficulties, struggles connecting to digital coaching sessions and complications advancing into the app after answering screening questions, were expressed.

Thought that the app was difficult to navigate and that you had to go back many times. – Study ID 156

A few users expressed low usability and visibility of the functions on their specific mobile phone model; meanwhile, others thought the appearance of the app was dull. These were said to hinder engagement. Moreover, several users voiced having difficulties understanding what needed to be done in the action plan and how the goal and activity setting worked. Therefore, the concept of the action plan was not clear to these users. Thus, it was voiced that the service needs to be optimised to become more user-friendly and clearer. Receiving technical support to navigate around the app was suggested, as well as a monthly calendar function to visualise goals and activities.

Various social and external factors were expressed as barriers of engagement. It was expressed that low engagement levels in the community function were a reason why users did not engage with it. The idea of engaging with others who were at different health levels was found to be stressful and also a reason why users did not engage in the community or coaching functions. Meanwhile, some expressed performance anxiety as a barrier of engaging with coaching. Moreover, several external factors represented key barriers of engagement for users, including work, summertime timing and one's life situation.

Several users felt overwhelmed when choosing which functions to use and what to focus on in the service and expressed a need to limit themselves. Some users realised that it was important to ‘go at your own pace’ and that everything could not be achieved at once.

Eh, but at the same time, you can’t focus on everything at the same time. – Study ID 158

Therefore, users expressed not being able to engage with all the functions in the service.

Product developers realised a need to motivate users to engage more and promote coaching within the app to increase engagement. Product developers also thought that what the service entails and provides needs to be clarified so that users can know what to expect from using the service and that this could boost motivation.

One thing we are looking at now is to even better and more clearly explain to the user before they step into the app, what they have to expect, how we help them and how the journey looks. – Product developer ID 1

### The service promoted health awareness and assisted behaviour change

All users expressed that the content inspired them to think about positive healthy thoughts and that the service promoted health awareness. The overarching health focus was appreciated, found to be unique and said to be healthy, natural, balanced and positive and reminded them of important aspects of health, which inspired a healthy mindset.

I thought it was great because it was really health. – Study ID 158

Users appreciated the inner self-focus message — that is to take care of oneself, find your own balance and feel good at your own level. It was also thought that the most essential elements for health were included in the service.

Users expressed that the coaching sessions stimulated healthy thoughts and provided support for how to complete goals and maintain behaviour change. It was also voiced that the service gave them a ‘kickstart’ and helped them go on the right path for behaviour change.

Yes, it gave me a bit of a kick in the butt. – Study ID 126

Some users acquired more structure and progress from the service. Moreover, the action plan and being able to go at your own pace were thought of as motivating factors for behaviour change. Several users mentioned that they had taken habits with them after using the service; however, it was voiced that users wanted to receive more behaviour change support in the service.

Coaches thought that the health message in the service was unique and the way forward for long-term health.

The app focus on the whole and includes mental balance which is unique with the app and hasn’t been seen before… this here is the way forward for long-term health, I think – Coach ID 2

Coaches believed that they helped users with their behaviour change and found that those who had multiple coaching sessions made progress.

Product developers voiced that the community and coaching functions, in particular, as well as being able to form trust with users by providing information based on research evidence to be important facilitators for behaviour change.

## Discussion

### Main findings

This feasibility study set out to investigate the acceptability, usability, engagement and optimisation of a mHealth service, which aims to promote and support healthy lifestyle behaviours. The quantitative score was found to be acceptable for acceptability. Quantitative and qualitative results suggested that the usability of LLA was not acceptable. Engagement in all functions reduced with time. Qualitative findings indicated that functions in LLA were appreciated and that LLA promoted health awareness and assisted in behaviour change. Moreover, qualitative findings have shed light on factors that influenced engagement, including numerous factors that hindered engagement. Strategies to optimise LLA were identified and included a need to increase personalisation, promote motivation and improve usability. The overall interpretation is that, although users appreciated using the service, the usability was not acceptable, several factors hindered engagement and therefore optimisations are needed for optimal use and to promote engagement.

### Comparison with previous literature

Users valued several functions of LLA, in particular, the action plan and coaching; which helps to explain the acceptable score for acceptability (3.3/5.0). Users enjoyed setting goals and activities in the action plan and found the action plan to be motivating for behaviour change. This can explain the relatively high quantitative engagement levels found for the action plan. The coaching sessions were also found to be important for behaviour change, linking to a previous finding, which found coaching to be an effective strategy for improving health behaviours.^16^ Both the action plan and coaching incorporated goal setting, self-monitoring and feedback BCTs; previously found to be valued by users of mHealth services.^15,18,49^ However, users voiced a desire for LLA to have more feedback and support for achieving goals, as well as the use of graphs to track progress; all of which have been voiced as attractive features by other end-users.^18^ Meanwhile, those who participated in coaching tended to have higher engagement in the action plan; indicating that coaching can be an important motivational factor for engagement and behaviour change. This links to a previous study which found that this type of blended approach, that is, the combination of goal setting, self-monitoring and coaching, was effective for PA behaviour change, in older adults.^50^

Other aspects of LLA were appreciated by users, including the library function and the overarching health focus. The content in the library function was found to be inspirational and suitable for the users’ health; which contributed to the positive score for information quality (4.0/5.0). By involving researchers in the service design, the content was vetted and made sure to follow evidence-based recommendations. Providing credible information has been shown to be favoured by other users and a facilitator of engagement.^15^ Users thought that the overarching health focus of LLA was unique and important for health. LLA's health message is to “feel good at your own level” with no mention of reducing calorie intake or striving to lose weight. Previous studies have found that goals relating to well-being compared to weight loss and body image goals have been more effective in increasing PA in middle-aged women.^51,52^ Therefore, this could be a useful consideration for future developments of mHealth services and could help to improve users’ self-image and facilitate a healthy balanced lifestyle.

However, not all users found LLA suitable and users made valuable suggestions for optimisation. The mixed qualitative findings on appreciation and suitability can provide reasoning for the just acceptable quantitative score for acceptability (3.3/5.0). A need for LLA to be more personalised was one of several suggestions voiced to facilitate engagement. Personalisation has been found to be effective in improving health behaviours.^16^ It has also been reported that users highly value the ability of mHealth services to allow for personalisation, in particular, the ability to receive personalised notifications and feedback.^18,49,53^ Other strategies to optimise the mHealth service, in this study, included more: push notifications, rewards and social interaction — including the possibility to invite friends and family members. The shortage of push notifications, rewards and possibilities to socially interact may help to explain why engagement in LLA reduced with time; as users may not have received enough motivation to continue to use the service. It may also explain why engagement levels in the coaching and community functions were not higher than that found. Previous findings have found push notifications^18,54^ and social interaction^53^ to be valued by some users but not suitable for all. These findings and that of this study connect to the notion that ‘one size does not fit all’; a key challenge in digital health promotion.^55^ Another key challenge in digital health promotion is overcoming high attrition rates.^20^ The results of this study suggest that allowing for notifications, rewards and social interaction and for these to be personalisable could facilitate prolonged engagement and reduce attrition. However, it is important to note that optimisations suggested by users in this study may only be suitable to users representative of this sample's demographics and who may already be motivated to maintain healthy lifestyle behaviours. Thus, optimisations identified in this study may not suit everyone and future digital health services should be tailored to their specific target population.

Insightful barriers of engagement were identified in this study, including usability difficulties, the struggle to limit oneself and contextual factors. The usability difficulties found help to explain the sub-acceptable quantitative score for usability (50/100). Having a user-friendly service is a pivotal facilitator of engagement for mHealth services.^18,49^ Moreover, providing navigation support can facilitate engagement,^54^ which was desired by users in this study. Users found it challenging to limit themselves and struggled to decide on what to focus on in the LLA app. It has previously been found that older adults can become overwhelmed by the choices presented in a mobile app, which can reduce acceptability.^56^ Limiting oneself may be why users in this study did not engage in all functions and why most users focused on just one area in LLA. Contextual factors such as summertime and work also represented barriers of engagement and may be why some users had limited engagement in LLA. Lack of time due to work and family responsibilities has previously been found to be a barrier for mid-life adults in engaging in healthy behaviours.^57^

Owing to a potential healthy selection bias, favouring already health-conscious individuals, users may have been at the maintenance stage in the Transtheoretical Model of Change.^37^ Therefore, users may have regularly engaged in healthy lifestyle behaviours and consequently had a diminished motivation to engage with LLA. Despite this, several users expressed acquiring a ‘kickstart’ in their behaviour change, which may help to explain why engagement levels were high at the beginning and dropped off with time. Previous research has shown that after habit formation and reaching competence in self-regulation, engagement in mHealth services reduces.^30^ As the intended app use for LLA varies among users, it is difficult to ascertain if the reduced engagement was indeed a positive sign due to users acquiring behaviour change or a negative sign due to factors that hindered engagement. It is believed that it can be a combination of both, however, given the numerous barriers to engagement identified and the quantitative scoring of usability, it is thought that reduced engagement was more due to the latter. Moreover, it is thought that to acquire long-term behaviour maintenance, users may need to engage in LLA regularly, yet this is subjective. A potential dose-response relationship may exist between levels of engagement and the long-term maintenance of behaviour; linking to the notion that “the more use, the better”.^29^ However, this relationship needs further exploring for LLA and is planned to be investigated in future studies.

Perspectives from product developers and coaches on operating and optimising LLA were explored. Coaches thought that the sessions went unexpectedly well and found the sessions to be valued by users, which seems to be in line with the users’ views. Coaches also deemed the digital format of the sessions to work well, which holds promise for the use of digital coaching in mHealth services. Product developers found it challenging to motivate users to engage and struggled to know how much push they should give. This links to the notion that the app can only do so much and ultimately one's inner motivation determines app use and behaviour change.^49^ Both product developers and coaches believed that the service provided a lot of value and possessed important functions for behaviour change, including the community function. However, given that the community function received less engagement than others, it is thought that this function will need to be optimised if it is to provide user value.

### Strengths and limitations

This study possesses several strengths. A mixed-methods approach was used to evaluate the feasibility of LLA, as suggested in Yardley et al., 2015^30^ and is often lacking in the field^58^ — the use of both methods allowed for a deeper evaluation. LLA was developed and tested using a co-development approach, involving stakeholders, which is recommended when assessing the feasibility of complex interventions^17^ and developing digital health interventions.^30^ The development of this service was also informed by several studies,^18,34,35^ carried out by members of the co-development team, that sought to identify users’ desires in mHealth services and support for behaviour change. This co-development and findings from these studies allowed evidence-based research to be incorporated into the service. However, due to time constraints, direct feedback from potential users was not incorporated into the co-development process, which would have strengthened the study further. Moreover, the perspectives of product developers and coaches were investigated in focus group discussions. Exploring the dialogues of those developing and working with mHealth services can inform on product development and app optimisation and provide an insight into what functions should be included to produce behaviour change. BCTs were linked with functions in the service, which has further provided insights into which BCTs can facilitate engagement and behaviour change. The coding of qualitative findings was checked by a researcher with experience in content analysis and a coding tree was provided, facilitating the judgment of credibility.^47^ Additionally, factors that facilitated or hindered engagement emerged in qualitative findings and were explored, covering another research gap.^59^

A limitation of the study was the use of convenience sampling rather than purposive. The sample was homogenous in characteristics and thus not representative of the general Swedish adult population. Moreover, due to the potential healthy selection bias, motivation to use LLA may have been lower compared to a sample with a higher need for behaviour change. This poses limitations for the transferability of the results to other populations. Men and people with lower education were identified as hard-to-reach groups and future studies should deploy a purposive sampling technique to acquire a heterogeneous sample. Additionally, the use of Facebook may have attracted a higher proportion of middle-aged adults and future studies should consider recruiting through other social media platforms, such as Instagram, to acquire a younger population.

Other limitations include the difficulty in determining whether reduced engagement was due to habit formation or barriers to technology use. Although the intended use of LLA has been defined and barriers to engagement were identified, it is difficult to interpret the exact reasons for reduced engagement. It is suggested that when users have different goals when using technology, intended use should be tailored to each individual — providing deeper insights into how using different functions can influence outcomes.^29^ Thus, applying this method of intended use could help to further understand the engagement patterns of LLA. Despite all users being invited to be part of the interviews, only eight users agreed to participate. However, it is thought that rich data was produced from these eight interviewees. The interview sample may have had more positive views than the general sample, representing a possible selection bias. Moreover, a product developer was present during the interviews with users and the focus group with coaches, which may have induced a positive response bias and influenced both users’ and coaches’ views. Nevertheless, users were informed to be as transparent as possible and to talk about both positive and negative experiences of using the service; which would help to inform on future optimisations. Additionally, the perspectives of product developers and coaches were included in the analyses. These could have been positively biased, but we intended to highlight their experiences and reflections on app optimisation. Only 15 users answered questions relating to acceptability and usability and at only one time point which limits the power of these findings. Furthermore, losing engagement data for the library function reduces the comprehensiveness of the engagement data and the validity of the qualitative findings for this function.

### Future perspectives

Suggestions for optimisation from users will be incorporated into future developments of LLA, as laid out in Yardley et al. (2015).^30^ This study has provided valuable insights into which components should be included when developing effective health-promoting mHealth services. This study also inspires future study designs and how to assess the acceptability, usability, engagement and optimisation of mHealth services. It is planned to carry out a feasibility study in a population of low socioeconomic position to uncover how this population perceives LLA and what optimisations are suggested to tailor the service to this target population. This work aims to contribute to reducing health inequities and making digital health services more suitable for everyone. Moreover, a large trial to test the effectiveness of LLA on multiple health outcomes is planned and engagement in LLA as a mediator for behaviour change will be investigated. This will help to see how much engagement is potentially needed to elicit changes in health outcomes, which could provide insights into how intended use is envisioned.

## Conclusion

Despite the incorporation of research-based evidence and behaviour theory, this pilot version of LLA needs to be optimised to improve usability, promote engagement and encourage long-term behaviour maintenance. Nevertheless, the health focus delivered in LLA was viewed positively and found to be unique. Moreover, LLA assisted users with their behaviour change by giving them a ‘kickstart’ in performing healthy lifestyle behaviours. This study has shown that even when a mHealth service is co-developed, based on research evidence and grounded in behaviour theory, feasibility testing and investigating users’ experiences are essential to develop suitable and effective mHealth services. The results of this study have further shed light on the all-importance of personalisation regarding behaviour change. With feasibility testing and investigating reasons for engagement, it is believed that mHealth services hold promise to make positive impacts on and support peoples’ healthy lifestyle behaviours.

## Supplemental Material

sj-docx-1-dhj-10.1177_20552076241247935 - Supplemental material for The acceptability, usability, engagement and optimisation of a mHealth service promoting healthy lifestyle behaviours: A mixed method feasibility study Supplemental material, sj-docx-1-dhj-10.1177_20552076241247935 for The acceptability, usability, engagement and optimisation of a mHealth service promoting healthy lifestyle behaviours: A mixed method feasibility study by Callum Regan, Phillip Von Rosen, Susanne Andermo, Maria Hagströmer, Unn-Britt Johansson and Jenny Rossen in DIGITAL HEALTH

sj-docx-2-dhj-10.1177_20552076241247935 - Supplemental material for The acceptability, usability, engagement and optimisation of a mHealth service promoting healthy lifestyle behaviours: A mixed method feasibility study Supplemental material, sj-docx-2-dhj-10.1177_20552076241247935 for The acceptability, usability, engagement and optimisation of a mHealth service promoting healthy lifestyle behaviours: A mixed method feasibility study by Callum Regan, Phillip Von Rosen, Susanne Andermo, Maria Hagströmer, Unn-Britt Johansson and Jenny Rossen in DIGITAL HEALTH

sj-docx-3-dhj-10.1177_20552076241247935 - Supplemental material for The acceptability, usability, engagement and optimisation of a mHealth service promoting healthy lifestyle behaviours: A mixed method feasibility study Supplemental material, sj-docx-3-dhj-10.1177_20552076241247935 for The acceptability, usability, engagement and optimisation of a mHealth service promoting healthy lifestyle behaviours: A mixed method feasibility study by Callum Regan, Phillip Von Rosen, Susanne Andermo, Maria Hagströmer, Unn-Britt Johansson and Jenny Rossen in DIGITAL HEALTH

sj-docx-4-dhj-10.1177_20552076241247935 - Supplemental material for The acceptability, usability, engagement and optimisation of a mHealth service promoting healthy lifestyle behaviours: A mixed method feasibility study Supplemental material, sj-docx-4-dhj-10.1177_20552076241247935 for The acceptability, usability, engagement and optimisation of a mHealth service promoting healthy lifestyle behaviours: A mixed method feasibility study by Callum Regan, Phillip Von Rosen, Susanne Andermo, Maria Hagströmer, Unn-Britt Johansson and Jenny Rossen in DIGITAL HEALTH

sj-docx-5-dhj-10.1177_20552076241247935 - Supplemental material for The acceptability, usability, engagement and optimisation of a mHealth service promoting healthy lifestyle behaviours: A mixed method feasibility study Supplemental material, sj-docx-5-dhj-10.1177_20552076241247935 for The acceptability, usability, engagement and optimisation of a mHealth service promoting healthy lifestyle behaviours: A mixed method feasibility study by Callum Regan, Phillip Von Rosen, Susanne Andermo, Maria Hagströmer, Unn-Britt Johansson and Jenny Rossen in DIGITAL HEALTH

sj-docx-6-dhj-10.1177_20552076241247935 - Supplemental material for The acceptability, usability, engagement and optimisation of a mHealth service promoting healthy lifestyle behaviours: A mixed method feasibility study Supplemental material, sj-docx-6-dhj-10.1177_20552076241247935 for The acceptability, usability, engagement and optimisation of a mHealth service promoting healthy lifestyle behaviours: A mixed method feasibility study by Callum Regan, Phillip Von Rosen, Susanne Andermo, Maria Hagströmer, Unn-Britt Johansson and Jenny Rossen in DIGITAL HEALTH
