# Supplementary material for: The acceptability, usability, engagement and optimisation of a mHealth service promoting healthy lifestyle behaviours: A mixed method feasibility study
Source: Digit Health. 2024 Apr 17;10:20552076241247935. doi: 10.1177/20552076241247935 (PMC11025415; doi:10.1177/20552076241247935)
Supplement: sj-docx-2-dhj-10.1177_20552076241247935 - Supplemental material for The acceptability, usability, engagement and optimisation of a mHealth service promoting healthy lifestyle behaviours: A mixed method feasibility study [file sj-docx-2-dhj-10.1177_20552076241247935.docx]

Behaviour change techniques incorporated into the functions of LLA

**LLA functions Behaviour change techniques included**

Action plan ***1. Goals and planning***
1.1 Goal setting (behaviour), 1.2 Problem solving, 1.3 Goal setting (outcome) 1.4 Action planning, 1.5 Review behaviour goals, 1.6 Discrepancy between current behaviour and goal, 1.7 Review outcome goals, 1.8 Behavioural contract, 1.9 Commitment,

***2. Feedback and monitoring***
2.2 Feedback on behaviour, 2.3 Self-monitoring of behaviour, 2.4 Self-monitoring of outcome(s) of behaviour, 2.7 Feedback on outcome(s) of behaviour,

***4. Shaping knowledge***
4.1 Instruction on how to perform a behaviour

***5. Natural consequences***
5.4 Monitoring of emotional consequenses

***6. Comparison of behaviour***
6.1 Demonstration of the behaviour

***8. Repetition and substitution***
8.1 Behavioural practice/rehearsal 8.2 Behaviour substitution, 8.4 Habit reversal, 8.7 Graded tasks

***9. Comparison of outcomes***
9.3 Comparative imagining of future outcomes

Feedback ***1. Goals and planning***

1.5 Review behaviour goals, 1.6 Discrepancy between current behaviour and goal, 1.7 Review outcome goals

***2. Feedback and monitoring***

2.2 Feedback on behaviour, 2.7 Feedback on outcome(s) of behaviour

***6. Comparison of behaviour***

6.2 Social comparison

***7. Associations***

7.1 Prompts/cues, 7.3 Reduce prompts/cues

***10. Reward and threat***

10.4 Social reward

***11. Regulation***

11.3 Conserving mental resources

***14. Scheduled consequences***

14.1 Anticipation of future rewards, 14.5 Rewarding completion,

***15. Self-belief***

15.1 Verbal persuasion about capability

Knowledge Library ***4. Shaping knowledge***

4.1 Instruction on how to perform a behaviour, 4.2 Information about antecedents

***5. Natural consequences***

5.1 Information about health consequenses, 5.6 Information about emotional consequences

***6. Comparison of behaviour***

6.1 Demonstration of the behaviour

***8. Repetition and substitution***

8.1 Behavioural practice/rehearsal, 8.2 Behaviour substitution, 8.3 Habit formation

***9. Comparison of outcomes***

9.1 Credible source

***11. Regulation***

11.2 Reduce negative emotions, 11.3 Conserving mental resources

***12. Antecedents***

12.1 Restructuring the physical environment, 12.2 Restructuring the social environment, 12.3 Avoidance/reducing exposure to cues for the behaviour, 12.4 Distraction, 12.6 Body changes

***13. Identity***

13.1 Identification as role model

***15. Self-belief***

15.1 Verbal persuasion about capability, 15.3 Focus on past success

Community ***1. Goals and planning***

1.2 Problem solving

***3. Social support***

3.1 Social support (unspecified), 3.2 Social support (practical), 3.3 Social support (emotional)

***5. Natural consequences***

5.1 Information about health consequenses, 5.6 Information about emotional consequences

***6. Comparison of behaviour***

6.1 Demonstration of the behaviour, 6.2 Social comparison, 6.3 Information about others' approval

***8. Repetition and substitution***

8.1 Behavioural practice/rehearsal, 8.2 Behaviour substitution

***9. Comparison of outcomes***

9.1 Credible source

***10. Reward and threat***

10.4 Social reward

***11. Regulation***

11.3 Conserving mental resources

***12. Antecedents***

12.1 Restructuring the physical environment, 12.2 Restructuring the social environment, 12.6 Body changes

***13. Identity***

13.1 Identification as role model, 13.5 Identity associated with changed behaviour

***15. Self-belief***

15.1 Verbal persuasion about capability, 15.3 Focus on past success

Coaching ***1. Goals and planning***

1.1 Goal setting (behaviour), 1.2 Problem solving, 1.3 Goal setting (outcome), 1.4 Action planning, 1.5 Review behaviour goals, 1.6 Discrepancy between current behaviour and goal, 1.8 Behavioural contract

***2. Feedback and monitoring***

2.2 Feedback on behaviour, 2.3 Feedback on outcome(s) of behaviour

***3. Social support***

3.1 Social support (unspecified), 3.2 Social support (practical), 3.3 Social support (emotional)

***8. Repetition and substitution***

8.2 Behaviour substitution, 8.3 Habit formation, 8.4 Habit reversal, 8.7 Graded tasks

***9. Comparison of outcomes***

9.1 Credible source, 9.2 Pros and cons

***10. Reward and threat***

10.4 Social reward

***14. Scheduled consequences***

14.1 Anticipation of future rewards

***15. Self-belief***

15.1 Verbal persuasion about capability, 15.3 Focus on past success
