# Supplementary material for: The acceptability, usability, engagement and optimisation of a mHealth service promoting healthy lifestyle behaviours: A mixed method feasibility study
Source: Digit Health. 2024 Apr 17;10:20552076241247935. doi: 10.1177/20552076241247935 (PMC11025415; doi:10.1177/20552076241247935)

Screenshots of the functions in LongLife Active®


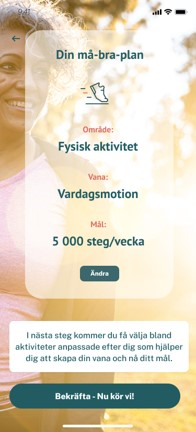

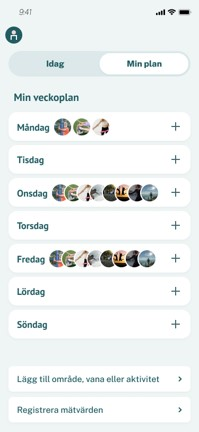
Action plan- goal setting within the physical activity area (5000 steps per week) on the left and weekly activity setting on the right

Community- the start page in the community function, from top to bottom: my groups, live events and news & inspiration.


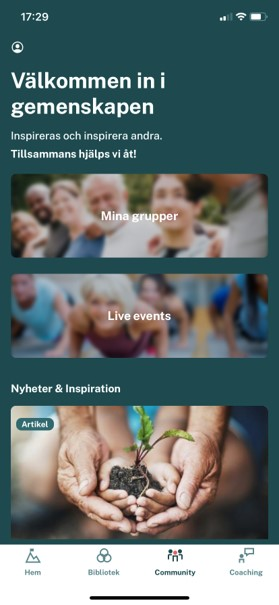


Coaching- start page for coaching and booking an individual coaching session

The knowledge library- the start page of the knowledge library, from top to bottom: training pass, recipes and meditation.


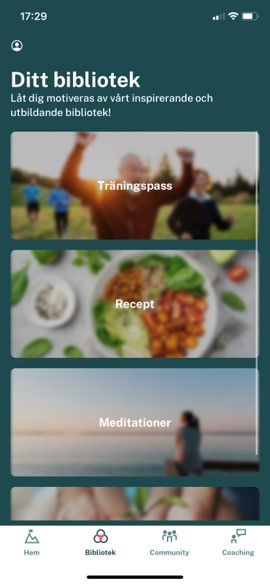


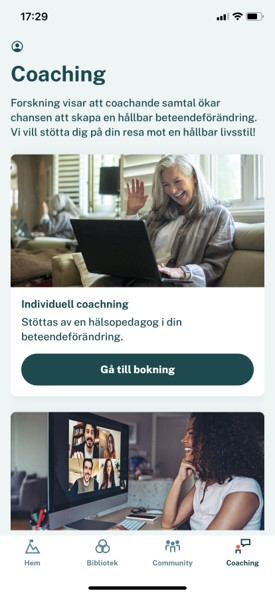

Supplement: sj-docx-3-dhj-10.1177_20552076241247935 - Supplemental material for The acceptability, usability, engagement and optimisation of a mHealth service promoting healthy lifestyle behaviours: A mixed method feasibility study [file sj-docx-3-dhj-10.1177_20552076241247935.docx]
