# Supplementary material for: The acceptability, usability, engagement and optimisation of a mHealth service promoting healthy lifestyle behaviours: A mixed method feasibility study
Source: Digit Health. 2024 Apr 17;10:20552076241247935. doi: 10.1177/20552076241247935 (PMC11025415; doi:10.1177/20552076241247935)
Supplement: sj-docx-5-dhj-10.1177_20552076241247935 - Supplemental material for The acceptability, usability, engagement and optimisation of a mHealth service promoting healthy lifestyle behaviours: A mixed method feasibility study [file sj-docx-5-dhj-10.1177_20552076241247935.docx]

SUS - A quick and dirty usability scale

John Brooke

Redhatch Consulting Ltd., 12 Beaconsfield Way, Earley, READING RG6 2UX

United Kingdom

email: [*john.brooke@redhatch.co.uk*](mailto:john.brooke@redhatch.co.uk)

# Abstract

*Usability does not exist in any absolute sense; it can only be defined with reference to particular contexts. This, in turn, means that there are no absolute measures of usability, since, if the usability of an artefact is defined by the context in which that artefact is used, measures of usability must of necessity be defined by that context too. Despite this, there is a need for broad general measures which can be used to compare usability across a range of contexts. In addition, there is a need for “quick and dirty” methods to allow low cost assessments of usability in industrial systems evaluation. This chapter describes the System Usability Scale (SUS) a reliable, low-cost usability scale that can be used for global assessments of systems usability.*

# Usability and context

Usability is not a quality that exists in any real or absolute sense. Perhaps it can be best summed up as being a general quality of the ***appropriateness to a purpose*** of any particular artefact. This notion is neatly summed up by Terry Pratchett in his novel “Moving Pictures”:

“ „Well, at least he keeps himself fit,‟ said the Archchancellor nastily. „Not like the rest of you fellows. I went into the Uncommon Room this morning and it was full of chaps snoring!‟

„That would be the senior masters, Master,‟ said the Bursar. „I would say they are supremely fit, myself.‟

„*Fit?* The Dean looks like a man who‟s swallered a bed!‟

„Ah, but Master,‟ said the Bursar, smiling indulgently, „the word “fit”,as I understand it, means

“appropriate to a purpose”, and I would say that the body of the Dean is supremely appropriate to the purpose of sitting around all day and eating big heavy meals.‟ The Dean permitted himself a little smile. “ (Pratchett, 1990)

In just the same way, the usability of any tool or system has to be viewed in terms of the context in which it is used, and its appropriateness to that context. With particular reference to information systems, this view of usability is reflected in the current draft international standard ISO 9241-11 and in the European Community ESPRIT project MUSiC (Measuring Usability of Systems in Context) (e.g., Bevan, Kirakowski and Maissel, 1991). In general, it is impossible to specify the usability of a system (i.e., its fitness for purpose) without first defining who are the intended users of the system, the tasks those users will perform with it, and the characteristics of the physical, organisational and social environment in which it will be used.

Since usability is itself a moveable feast, it follows that measures of usability must themselves be dependent on the way in which usability is defined. It is possible to talk of some general classes of usability measure; ISO 9241-11 suggests that measures of usability should cover


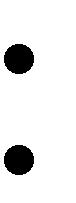

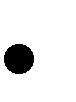
 effectiveness ( the ability of users to complete tasks using the system, and the quality of the output of those tasks),

efficiency ( the level of resource consumed in performing tasks) satisfaction (users’ subjective reactions to using the system).

However, the precise measures to be used within each of these classes of metric can vary widely. For example, measures of effectiveness are very obviously determined by the types of task that are carried out with the system; a measure of effectiveness of a word processing system might be the number of letters written, and whether the letters produced are free of spelling mistakes. If the system supports the task of controlling an industrial process producing chemicals, on the other hand, the measures of task completion and quality are obviously going to reflect that process.

A consequence of the context-specificity of usability and measures of usability is that it is very difficult to make comparisons of usability across different systems. Comparing usability of different systems intended for different purposes is a clear case of “comparing apples and oranges” and should be avoided wherever possible. It is also difficult and potentially misleading to generalise design features and experience across systems; for example, just

because a particular design feature has proved to be very useful in making one system usable does not necessarily mean that it will do so for another system with a different group of users doing different tasks in other environments.

If there is an area in which it is possible to make more generalised assessments of usability, which could bear cross-system comparison, it is the area of subjective assessments of usability. Subjective measures of usability are usually obtained through the use of questionnaires and attitude scales, and examples exist of general attitude scales which are not specific to any particular system (for example, CUSI (Kirakowski and Corbett, 1988)).

# Industrial usability evaluation

The demands of evaluating usability of systems within an industrial context mean that often it is neither cost-effective nor practical to perform a full-blown context analysis and selection of suitable metrics. Often, all that is needed is a general indication of the overall level of usability of a system compared to its competitors or its predecessors. Equally, when selecting metrics, it is often desirable to have measures which do not require vast effort and expense to collect and analyse data.

These sorts of considerations were very important when, while setting up a usability engineering programme for integrated office systems engineering with Digital Equipment Co. Ltd, a need was identified for a subjective usability measure. The measure had to be capable of being administered quickly and simply, but also had to be reliable enough to be used to make comparisons of user performance changes from version to version of a software product.

The need for simplicity and speed came from the evaluation methods being used; users from customer sites would either visit a human factors laboratory, or a travelling laboratory would be set up at the customer site. The users would then work through evaluation exercises lasting between 20 minutes and an hour, at the end of which a subjective measure of system usability would be collected. As can be imagined, after this period of time, users could be very frustrated, especially if they had encountered problems, since no assistance was given. If they were then presented with a long questionnaire, containing in excess of 25 questions it was very likely that they would not complete it and there would be insufficient data to assess subjective reactions to system usability.

# SUS - the System Usability Scale

In response to these requirements, a simple usability scale was developed. The System Usability Scale (SUS) is a simple, ten-item scale giving a global view of subjective assessments of usability.

SUS is a *Likert scale*. It is often assumed that a Likert scale is simply one based on forced- choice questions, where a statement is made and the respondent then indicates the degree of agreement or disagreement with the statement on a 5 (or 7) point scale. However, the construction of a Likert scale is somewhat more subtle than this. Whilst Likert scales are presented in this form, the statements with which the respondent indicates agreement and disagreement have to be selected carefully.

The technique used for selecting items for a Likert scale is to identify examples of things which lead to extreme expressions of the attitude being captured. For instance, if one was interested in attitudes to crimes and misdemeanours, one might use serial murder and parking offences as examples of the extreme ends of the spectrum. When these examples have been selected, then a sample of respondents is asked to give ratings to these examples across a wide pool of potential questionnaire items. For instance, respondents might be asked to respond to statements such as “hanging’s too good for them”, or “I can imagine myself doing something like this”.

Given a large pool of such statements, there will generally be some where there is a lot of agreement between respondents. In addition, some of these will be ones where the statements provoke extreme statements of agreement or disagreement among all respondents. It is these latter statements which one tries to identify for inclusion in a Likert scale, since, we would hope that, if we have selected suitable examples, there would be general agreement of extreme attitudes to them. Items where there is ambiguity are not good discriminators of attitudes. For instance, while one hopes that there would be a general, extreme disagreement that “hanging’s too good” for those who perpetrate parking offences, there may well be less agreement about applying this statement to serial killers, since opinions differ widely about the ethics and efficacy of capital punishment.

SUS was constructed using this technique. A pool of 50 potential questionnaire items was assembled. Two examples of software systems were then selected (one a linguistic tool aimed at end users, the other a tool for systems programmers) on the basis of general agreement that one was “really easy to use” and one was almost impossible to use, even for highly technically skilled users. 20 people from the office systems engineering group, with occupations ranging from secretary through to systems programmer then rated both systems against all 50 potential questionnaire items on a 5 point scale ranging from “strongly agree” to “strongly disagree”.

The items leading to the most extreme responses from the original pool were then selected. There were very close intercorrelations between all of the selected items (± 0.7 to ± 0.9). In addition, items were selected so that the common response to half of them was strong agreement, and to the other half, strong disagreement. This was done in order to prevent response biases caused by respondents not having to think about each statement; by alternating positive and negative items, the respondent has to read each statement and make an effort to think whether they agree or disagree with it.

The System Usability Scale is shown in the next section of this chapter. It can be seen that the selected statements actually cover a variety of aspects of system usability, such as the need for support, training, and complexity, and thus have a high level of face validity for measuring usability of a system.

# System Usability Scale

© Digital Equipment Corporation, 1986.

1. I think that I would like to use this system frequently

2. I found the system unnecessarily complex

3. I thought the system was easy to use

4. I think that I would need the support of a technical person to be able to use this system

5. I found the various functions in this system were well integrated

6. I thought there was too much inconsistency in this system

7. I would imagine that most people would learn to use this system very quickly

8. I found the system very cumbersome to use

9. I felt very confident using the system

10. I needed to learn a lot of things before I could get going with this system

Strongly Strongly

disagree agree

|  |  |  |  |  |
| --- | --- | --- | --- | --- |

| 1 | 2 | 3 | 4 | 5 |
| --- | --- | --- | --- | --- |
|  |  |  |  |  |
| 1 | 2 | 3 | 4 | 5 |

|  |  |  |  |  |
| --- | --- | --- | --- | --- |

| 1 | 2 | 3 | 4 | 5 |
| --- | --- | --- | --- | --- |
|  |  |  |  |  |
| 1 | 2 | 3 | 4 | 5 |

|  |  |  |  |  |
| --- | --- | --- | --- | --- |

1 2 3 4 5

|  |  |  |  |  |
| --- | --- | --- | --- | --- |

1 2 3 4 5

|  |  |  |  |  |
| --- | --- | --- | --- | --- |

| 1 | 2 | 3 | 4 | 5 |
| --- | --- | --- | --- | --- |
|  |  |  |  |  |
| 1 | 2 | 3 | 4 | 5 |

|  |  |  |  |  |
| --- | --- | --- | --- | --- |

| 1 | 2 | 3 | 4 | 5 |
| --- | --- | --- | --- | --- |
|  |  |  |  |  |
| 1 | 2 | 3 | 4 | 5 |

# Using SUS

The SU scale is generally used after the respondent has had an opportunity to use the system being evaluated, but before any debriefing or discussion takes place. Respondents should be asked to record their immediate response to each item, rather than thinking about items for a long time.

All items should be checked. If a respondent feels that they cannot respond to a particular item, they should mark the centre point of the scale.

# Scoring SUS

SUS yields a single number representing a composite measure of the overall usability of the system being studied. Note that scores for individual items are not meaningful on their own.

To calculate the SUS score, first sum the score contributions from each item. Each item's score contribution will range from 0 to 4. For items 1,3,5,7,and 9 the score contribution is the scale position minus 1. For items 2,4,6,8 and 10, the contribution is 5 minus the scale position. Multiply the sum of the scores by 2.5 to obtain the overall value of SU.

SUS scores have a range of 0 to 100.

The following section gives an example of a scored SU scale.

# System Usability Scale

© Digital Equipment Corporation, 1986.

1. I think that I would like to use this system frequently

2. I found the system unnecessarily complex

3. I thought the system was easy to use

4. I think that I would need the support of a technical person to be able to use this system

5. I found the various functions in this system were well integrated

6. I thought there was too much inconsistency in this system

7. I would imagine that most people would learn to use this system very quickly

8. I found the system very cumbersome to use

9. I felt very confident using the system

10. I needed to learn a lot of things before I could get going with this system

**Total score = 22**

**SUS Score = 22 *22.5 = 55**

Strongly Strongly

disagree agree

## 4

|  |  |  |  | 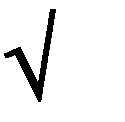 |
| --- | --- | --- | --- | --- |

1 2 3 4 5

## 1

|  |  |  | 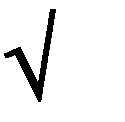 |  |
| --- | --- | --- | --- | --- |

1 2 3 4 5

## 1

|  | 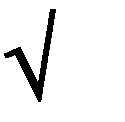 |  |  |  |
| --- | --- | --- | --- | --- |

1 2 3 4 5

## 4

| 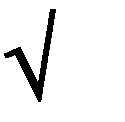 |  |  |  |  |
| --- | --- | --- | --- | --- |

1 2 3 4 5

## 1

|  | 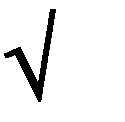 |  |  |  |
| --- | --- | --- | --- | --- |

1 2 3 4 5

## 2

|  |  | 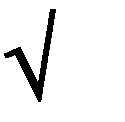 |  |  |
| --- | --- | --- | --- | --- |

1 2 3 4 5

## 1

|  | 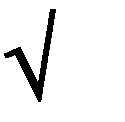 |  |  |  |
| --- | --- | --- | --- | --- |

1 2 3 4 5

## 1

|  |  |  | 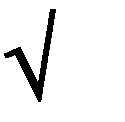 |  |
| --- | --- | --- | --- | --- |

1 2 3 4 5

## 4

|  |  |  |  | 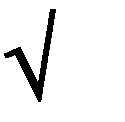 |
| --- | --- | --- | --- | --- |

1 2 3 4 5

## 3

|  | 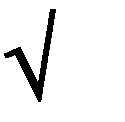 |  |  |  |
| --- | --- | --- | --- | --- |

1 2 3 4 5

# Conclusion

SUS has proved to be a valuable evaluation tool, being robust and reliable. It correlates well with other subjectives measures of usability (eg., the general usability subscale of the SUMI inventory developed in the MUSiC project (Kirakowski, personal communication)). SUS has been made freely available for use in usability assessment, and has been used for a variety of research projects and industrial evaluations; the only prerequisite for its use is that any published report should acknowledge the source of the measure.

# Acknowledgements

SUS was developed as part of the usability engineering programme in integrated office systems development at Digital Equipment Co Ltd., Reading, United Kingdom.

# References

Bevan, N, Kirakowski, J and Maissel, J, 1991, What is Usability?, in H.-J. Bullinger, (Ed.). *Human Aspects in Computing: Design and use of interactive systems and work with terminals*, Amsterdam: Elsevier.

Kirakowski, J and Corbett, M, 1988, Measuring User Satisfaction, in D M Jones and R Winder (Eds.) *People and Computers IV*. Cambridge: Cambridge University Press.

Pratchett, T., 1990 *Moving Pictures*. London: Gollancz
