# Supplementary material for: The acceptability, usability, engagement and optimisation of a mHealth service promoting healthy lifestyle behaviours: A mixed method feasibility study
Source: Digit Health. 2024 Apr 17;10:20552076241247935. doi: 10.1177/20552076241247935 (PMC11025415; doi:10.1177/20552076241247935)
Supplement: sj-docx-6-dhj-10.1177_20552076241247935 - Supplemental material for The acceptability, usability, engagement and optimisation of a mHealth service promoting healthy lifestyle behaviours: A mixed method feasibility study [file sj-docx-6-dhj-10.1177_20552076241247935.docx]

Coding tree- examples of meaning units and codes from themes.

| Theme | Sub-theme | Code | Exemplar meaning units |
| --- | --- | --- | --- |
| One size does not fit all; perspectives on appreciation, suitability and personalisation |  | The food content was good and suitable | *“I think that when it applies to the food that was really good for me”- Study ID 104* |
| One size does not fit all; perspectives on appreciation, suitability and personalisation |  | Inspiring new things | *“You became inspired and thought that this here can be done and saw what little more there is”…“It’s very, it opened the eyes a bit to what you can do”- Study ID 124* |
| One size does not fit all; perspectives on appreciation, suitability and personalisation |  | Three codes:  Coaching for level setting  Coaching for prioritising  Coaching was important | *“To be able to talk to another person about where to set your level, and channel it a bit. What is it you need and what do you want with it. I felt that was really important”- Study ID 154* |
| One size does not fit all; perspectives on appreciation, suitability and personalisation |  | Disappointed about the lack of inspiration | *“I didn’t find the inspiration, so what I hoped for was that I would get inspiration from this, your app”- Study ID 156* |
| One size does not fit all; perspectives on appreciation, suitability and personalisation |  | It is important to have a variation | *“We are already very mobile-intensive in today’s situation, so therefore I think it is good to have a little variation”- Study ID 104* |
| One size does not fit all; perspectives on appreciation, suitability and personalisation |  | More personalisation | *“I know I got some notifications like this and that, but maybe you should have it so that you can choose your level yourself… “Because then you feel that it is even more personalised”- Study ID 141* |
| One size does not fit all; perspectives on appreciation, suitability and personalisation |  | A lot of value provided | *“It feels like we are providing so much value within the app, I think, it is difficult to see what we feel is the most powerful of those. If I would pick one, I would say the coaching sessions would be the most powerful ones”- Product developer ID 1* |
| Influencers of engagement | Facilitators of engagement | Great foundation and structure | *“I think you have made a great foundation and built it up so to speak”- Study ID 126* |
| Influencers of engagement | Facilitators of engagement | The sessions feel the best when a relationship and trust is formed | *“The conversations that I feel have worked best for me are when I feel like we have some type of relationship. Eh, and that I feel that the person has trust in me, eh, and feels safe and... dares to reflect and dares to open up”- Coach ID 1* |
| Influencers of engagement | Facilitators of engagement | Reminders are needed | *“Think there should be a function in the app that reminds you that "now is the time" to register or accomplish today's goals”-* *Study ID 135* |
| Influencers of engagement | Facilitators of engagement | Share the journey | *“So that you have at least someone you know and that you can do the journey together with”- Study ID 154* |
| Influencers of engagement | Barriers of engagement | Concept confusion | *“So, I can’t even say that I understood that I would create an action plan”- Study ID 158* |
| Influencers of engagement | Barriers of engagement | Limited myself | *“It was quite difficult since I would have very much liked to go down that track also, but I realised I limited myself”- Study ID 124* |
| Influencers of engagement | Barriers of engagement | Summer as a barrier | *“It was a bit of summer, and it was a bit, you travelled more, and you had a bit of a split vision of what to bring with you and how to get into this here”- Study ID 141* |
| The service promoted health awareness and assisted behaviour change |  | Really health | *“I thought it was great because it was really health”- Study ID 158* |
| The service promoted health awareness and assisted behaviour change |  | Got more structure and a kickstart | *“For my part I have gained more structure on food and exercise. Even the mental calmness aspect has given me a ‘kick’”- Study ID 120* |
| The service promoted health awareness and assisted behaviour change |  | Continuation of habits | *“I will continue with my new eating habits thanks to the app/study. Exercise (walk and run), think about sleep and also plan social activities”-* *Study ID 104* |
| The service promoted health awareness and assisted behaviour change |  | The health focus is unique and the way for long-term health | *“The app focus on the whole and includes mental calmness which is unique with the app and hasn’t been seen before”…“this here is the way forward for long-term health, I think”- Coach ID 2* |
